# Supplementary material for: Development of Palm Fatty Acid Distillate-Containing Medium for Biosurfactant Production by Pseudomonas sp. LM19
Source: Molecules. 2019 Jul 18;24(14):2613. doi: 10.3390/molecules24142613 (PMC6680552; doi:10.3390/molecules24142613)
Supplement: Supplementary file 1 [file molecules-24-02613-s001.pdf]

Table S1. Analysis of variance (ANOVA) of the regression model from the PBD for medium components contribution to a) cell growth and b) maximum biosurfactant productivity

(a)

Source	DF*	SS*	MS*	F-value	ρ-value
Model	3	67.34	22.45	18.45	0.0003
A: KH_2PO_4	1	13.65	13.65	11.22	0.0085
E: Yeast extract	1	39.60	39.60	32.56	0.0003
G: Na-EDTA	1	14.08	14.08	11.58	0.0078
Residual	9	10.95	1.22		
Total	12	78.29			

Notes: DF, degrees of freedom; SS, sum of squares; MS, mean sum of squares.

S: 1.10, R^2: 0.8602, R^2 (pred): 0.8135, R^2 (adj): 0.7004

(b)

Source	DF*	SS*	MS*	F-value	ρ-value
Model	3	0.099	0.033	18.67	0.0003
A: KH_2PO_4	1	0.027	0.027	15.50	0.0034
C: $MgSO_4.7H_2O$	1	0.049	0.049	27.94	0.0005
E: Yeast extract	1	0.023	0.023	13.01	0.0057
Residual	9	0.016	1.77×10^{-3}		
Total	12	0.11			

Notes: DF, degrees of freedom; SS, sum of squares; MS, mean sum of squares.

S: 0.042, R^2: 0.8615, R^2 (pred): 0.8154, R^2 (adj): 0.7111

Table S2. Analysis of variance (ANOVA) of the regression model from the CCD for medium components contribution to a) cell growth and b) biosurfactant productivity in uncoded units

(a)

Source	*DF	*SS	*MS	F-value	ρ-value
Block	2	0.45	0.23		
Model	9	98.91	10.99	29.94	0.0001
C:Yeast extract	1	2.13	2.13	5.82	0.0204
D: Sodium-EDTA	1	2.27	2.27	6.19	0.070
E: $MgSO_4.7H_2O$	1	5.15	5.15	14.02	0.0006
C*D: Yeast extract*Sodium-EDTA	1	1.71	1.71	4.66	0.0367
A^2: PFAD*PFAD	1	16.75	16.75	45.63	0.0001
B^2: KH_2PO_4*KH_2PO_4	1	5.70	5.70	15.52	0.0003
C^2: Yeast extract*Yeast extract	1	44.70	44.70	121.78	0.0001
D^2: Sodium-EDTA*Sodium-EDTA	1	7.44	7.44	20.27	0.0001
E^2: $MgSO_4.7H_2O$* $MgSO_4.7H_2O$	1	32.06	32.06	87.34	0.0001
Residual	41	15.05	0.37		
Lack of Fit	33	12.86	0.39	1.43	0.3119
Pure Error	8	2.19	0.27		
Total	52	114.42			

Notes: DF, degrees of freedom; SS, sum of squares; MS, mean sum of squares

S: 0.61, R^2: 0.8679, R^2 (pred): 0.7369, R^2 (adj): 0.8389

(b)

Source	*DF	*SS	*MS	F-value	ρ-value
Block	2	2.58×10^{-3}	1.29×10^{-3}		
Model	9	0.40	0.045	19.39	0.0001
A: PFAD	1	0.039	0.039	16.92	0.0002
D: Sodium-EDTA	1	0.026	0.026	11.46	0.0016
E: $MgSO_4.7H_2O$	1	6.91×10^{-3}	6.91×10^{-3}	3.00	0.0905
A*B: PFAD*KH_2PO_4	1	9.61×10^{-3}	9.61×10^{-3}	4.18	0.0474
A^2: PFAD*PFAD	1	0.025	0.12	50.79	0.0019
B^2: KH_2PO_4* KH_2PO_4	1	0.11	0.025	11.06	0.0001
C^2: Yeast extract*Yeast extract	1	0.019	0.11	48.80	0.0066
D^2: Na-EDTA*Na-EDTA	1	0.12	0.019	8.19	0.0001
E^2: $MgSO_4.7H_2O$* $MgSO_4.7H_2O$	1	0.40	0.12	51.14	0.0001
Residual	41	0.094	2.30×10^{-3}		
Lack of Fit	33	0.086	2.60×10^{-3}	2.44	0.0935
Pure Error	8	8.52×10^{-3}	1.07×10^{-3}		
Total	52	0.50			

Notes: DF, degrees of freedom; SS, sum of squares; MS, mean sum of squares

S: 0.048, R^2: 0.8097, R^2 (pred): 0.6877, R^2 (adj): 0.7680
